# Differentiation of human embryonic germ cells and transplantation in rats with acute myocardial infarction

**DOI:** 10.3892/etm.2014.1474

**Published:** 2014-01-03

**Authors:** SHUICHANG YU, YANBO ZHU, FANG LI, YUJUAN ZHANG, CHUNLIN XIA

**Affiliations:** 1 Department of Histology and Embryology, Medical College of Soochow University, Suzhou 215123, P.R. China; 2 Department of Oncology, The First Affiliated Hospital of Soochow University, Suzhou 215006, P.R. China; 3 Boxi Institute of Clinical Anatomy and Cytoneurobiology Laboratory, Medical College of Soochow University, Suzhou 215123, P.R. China

**Keywords:** human embryonic germ cell, cell transplantation, myocardial infarction

## Abstract

Human embryonic germ cells (hEGCs) are stem cells cultured from primordial germ cells, which reside in human embryonic genital ridges *in vivo*. In this study, hEGCs were induced to differentiate into cardiomyocytes by treatment with ascorbic acid *in vitro* and the effects of hEGC transplantation on rat models of acute myocardial infarction (AMI) were investigated. hEGCs were incubated with differentiation medium containing ascorbic acid at various concentrations. Levels of GATA-4 expression were measured to identify the optimal concentration of the inductor. Immunofluorescence microscopy was used to detect the expression of Cx43 on the induced cells. The hEGCs were injected into the myocardium of rats with AMI. The expression levels of MAB1281 and GATA-4 were used to indicate the survival, migration, distribution and differentiation of transplanted cells. The results revealed the positive expression of GATA-4, Cx43 and cardiac troponin T (cTnT) in differentiated cells, and immunocytochemistry showed that transplanted cells highly expressed GATA-4 and MAB1281. hEGCs were successfully induced to differentiate into cardiomyocytes by ascorbic acid in optimal concentrations *in vitro* and the transplanted hEGCs survived and differentiated into cardiomyocytes.

## Introduction

Acute myocardial infarction (AMI) is an issue worldwide, which severely jeopardises human health. The majority of patients who suffer from AMI succumb to its complications, such as heart failure and arrhythmias. The mechanisms involved in repairing the infarcted zone remain unknown. Cell therapy of AMI involves transplanting various types of stem cells that have the potential to differentiate in the infarcted zone, where they proliferate and differentiate into cardiomyocytes, thus improving heart function (1,2).

Embryonic stem cells (ESCs) have the ability to respond to environmental demands. Previous studies have shown that retinoic acid induces the differentiation of ESCs into cardiomyocytes. Inductors also include dimethyl sulphoxide (DMSO), cAMP, neuregulin and deoxycytidine (3–6). Rajasingh *et al* found that leukaemia inhibitory factor (LIF) and bone morphogenetic protein 2 (BMP-2) were able to synergistically enhance the cardiomyocyte differentiation of transplanted stem cells, whereas DMSO or retinoic acid were not (7). In addition to using inductors, investigators have precipitated the differentiation of ESCs into cardiomyocytes naturally. Kehat *et al* observed that cardiomyocytes differentiated from ESCs have the characteristics of myocardial electrophysiology and surface proteins. The authors transplanted these cardiomyocytes into the infarcted zone of a pig and found that these cells integrated with the receptor cardiomyocytes, and the heart began to beat again (8).

Human embryonic germ cells (hEGCs) are stem cells cultured from primordial germ cells, which reside in human embryonic genital ridges *in vivo*. They are similar to human embryonic stem cells (hESCs), which form the inner cell mass, in biological characteristics. Our laboratory has established a system for obtaining hEGCs through tissue culturing. The study of hESC transplantation for the treatment of AMI started worldwide >10 years ago; however, the use of hEGCs as a transplantable cell source for treating AMI has not, to the best of our knowledge, been reported.

In the present study, ascorbic acid was used to induce hEGCs to differentiate into cardiomyocytes *in vitro* with the aim of identifying a simple and high-performance empirical method for promoting hEGC differentiation. In addition, hEGCs were injected into the infarcted zone in rat models of AMI in order to observe and identify the survival and differentiation of hEGCs, thereby providing experimental support for myocardiac tissue engineering research and the treatment of AMI by stem cell transplantation.

## Materials and methods

### Main reagents

The reagents used in this study were as follows: Dulbecco’s modified Eagle’s Medium (DMEM; Gibco-BRL, Carlsbad, CA, USA; high glucose); fetal calf serum (FCS; Hyclone, Beijing, China); basic fibroblast growth factor (bFGF), LIF, ascorbic acid, and Cx43 and cardiac troponin T (cTnT) mouse anti-human monoclonal antibodies (R&D Systems, Shanghai, China); FITC-labeled rabbit anti-mouse secondary antibody (Wuhan Boster Biological Technology, Ltd., Wuhan, China); MAB1281 mouse anti-human nuclei monoclonal antibody (Chemichon, Temecula, CA, USA); rabbit anti-GATA-4 (Wuhan Boster Biological Technology, Ltd.); and EnVision™ Detection kit [GK50075; comprising ChemMate™ DAKO EnVision™/HRP, Rabbit/Mouse(ENV); ChemMate™ Substrate Buffer and ChemMate™ 3,3′-diaminobenzidine (DAB) + Chromogen; Genentech, Inc., San Francisco, CA, USA]; Hoechst33342Cell Staining solution (Biohao, Beijing, China).

### Source of embryos

Human embryos aborted at 5–10 weeks, were collected from The First and Second Hospitals Affiliated to Soochow University (Suzhou, China) and the Maternal and Child Health Center (Suzhou, China), with the permission from pregnant women and the Morality Committee. All the embryos were intact and not severely contaminated.

### Cell culture and proliferation of hEGCs

The gonadal ridges of 5–10-week-old embryos were cultured as explant tissue *in vitro*, in high glucose DMEM supplemented with 15% FCS; LIF and bFGF were also added. The hEGCs and cell cultures were gained from the surface of the homologous human embryonic fibroblast, which was used as the feeder. After 8 days of primary culture, the hEGCs were digested and subcultured to passage 4.

### Formation of embryoid bodies (EBs) by suspension culture

The subconfluent cells were selected for digestion into a single cell suspension, then plated onto a gelatin (0.1%)-coated 100-mm Petri dish at 37ºC in humid air with 5% CO_2_ for 1 h to remove fibroblasts. Briefly, 1×10^5^ cells in 1 ml cultivation medium containing 20% FCS were suspended in bacteriological plates for 5 days. The medium was replaced every other day.

### Differentiation of hEGCs into cardiomyocytes

After suspension for 5 days, EBs were separately plated onto a gelatin (0.1%)-coated 24-well plate and various concentrations (0.01, 0.05, 0.1 and 0.2 mg/ml) of ascorbic acid were added. The medium was replaced with fresh medium every other day.

### Immunocytochemistry

Cells were collected at 1, 2, 3 and 4 weeks of induction and fixed in 4% paraformaldehyde, quenched with 3% hydrogen peroxide and non-specific background was blocked with a serum-free blocking solution. Primary antibodies were diluted in dilution buffer and incubated with the cells for 30 min at room temperature. Cells were washed in phosphate-buffered saline and a polymer-linked peroxidase-labeled secondary antibody was added for 30 min at room temperature. Cells were then stained with DAB and haematoxylin, fixed with neutral gum and photographed. Cardiomyocytes were identified immunocytochemically using anti-GATA-4 and anti-cTnT antibodies. Controls underwent staining without the primary antibody.

### Statistical analysis

Conversion rate of cardiac-like cells: three visual fields of the cell creep slices induced by the inductors were recorded. The total cell count and the count of cells positive for GATA-4 were recorded under a light microscope with a magnification of ×200 (Olympus, Shanghai, China). The conversion rate of hEGC differentiation into cardiac-like cells in the three fields was calculated. Results are expressed as the means ± standard deviation and statistically evaluated by Student’s t-test. P<0.05 was considered to indicate a statistically significant difference.

### Immunofluorescence

Cells were collected following induction for 2 weeks, and the expression levels of myocardial connectin Cx43 protein and cardiac troponin (cTnT) were detected by immunofluorescence. Cells were blocked by treatment with cold methanol in PBS for 30 min. After rinsing three times in PBS for 5 min each, rat anti-human (1:50) antibody diluted solution was added overnight at 4ºC. Cells were rinsed three times in PBS for 5 min each. Subsequently, the cells were incubated with FITC-labeled rabbit anti-mouse secondary antibody at a dilution of 1:50 in PBS for 1 h at room temperature. Cells were rinsed three times in PBS for 5 min each. Cells were then covered with 50% buffered glycerol and observed under a fluorescence microscope (Olympus).

### Experimental animals and grouping

Forty Sprague-Dawley rats, of clean grade and either gender (weight, 200–250 g) were provided by the Experimental Animal Center of Soochow University (Suzhou, China). These rats were randomly divided into two groups: The experimental group (n=28) and the control group (n=12). In the experimental group, hEGCs were injected into the edge of the infarcted zone of the rat models with AMI. Four points were selected and 12 μl cell suspension was injected into each point. In the control group, the same process was followed but only PBS was injected into the selected points to serve as the negative control.

### Preparation of the cells for transplantation

One hour prior to transplantation, the hEGC colony subcultured to the fourth passage was extracted with aseptic inoculating needles, and digested with 0.25% trypsinase and 0.02% EDTA. When cytoplasm recovery occurred and the cell colonies were scattered, blood serum medium was added to stop the trypsinization, and then a pipette was used to blow air across the cells and to strike the bottom of the bottle gently and repeatedly until the cells formed a steady cell suspension. The cells were centrifuged for 5 min at 1,000 × g, washed with sterile PBS and then blown on and struck until the cells were well distributed for cell counting. The cells were taken up with a microinjector at a concentration of 1×10^6^ ml (50 μl).

### Establishment of the rat model of myocardial infarction

The surgery group: The AMI model was established by ligation of the left anterior descending branch of the coronary artery. Rats were anesthetized with an intraperitoneal injection of 4% chloral hydrate and, following disinfection of the surgical area, tracheal intubation was performed and mechanical ventilation was initiated. With monitoring by electrocardiography (CONTEC), the thoracic cavity was opened from the left sternal border in the third or fourth intercostal muscles and the cardiac pericardium was opened until the heart was totally exposed. The left anterior descending branch of the coronary artery was ligated with 5.0 surgical silk sutures. Needle-shaped electrodes were attached under the four limbs. Lead II electrocardiograms (ECGs) were recorded. The myocardial infarction model was established until persistent ST elevation on the ECG indicative of myocardial infarction was observed. After the surgery, 400,000 units penicillin was injected into the rats in case of infection. The rats recovered at 30ºC. Strict aseptic conditions were maintained during the whole process; therefore, dermal sutures were unnecessary.

The sham surgery group: Between the lower margin of the left atrial appendage and pulmonary conus, namely the left anterior descending branch of the coronary artery, a non-invasive suture needle was used to perforate the lower part of the blood vessel without ligature. Other procedures were similar to those in the surgery group.

### Transplantation of hEGCs following myocardial infarction

The prepared hEGC suspension was directly injected into the infarcted zone in the heart of each rat. Under direct vision, after the ligature was removed from the anterior descending coronary artery, hEGCs were injected into the edge of the infarcted zone of the heart. Four points were selected and 12 μl cell suspension with a concentration of 1×10^6^ ml was injected into each. In the control group, the same process was followed, but only PBS was injected into the selected points to serve as the negative control. The pectus was closed and mechanical ventilation was maintained until spontaneous respiration occurred.

### Immunohistochemistry

One day and 1, 2 and 4 weeks following the transplantation, seven rats in the experimental group and three in the control group were selected. The rats were anaesthetised with an intraperitoneal injection of 4% hydral and their hearts were removed and washed in PBS. The left and right atria of the heart together with the right ventricle were eliminated, the left ventricle was fixed with 4% paraformaldehyde and embedded in paraffin for serial sectioning. Primary antibodies against MAB1281 (1:100) and GATA-4 (1:100), as well as universal secondary antibodies were adopted in a 2-step immunohistochemical method (5). Cells were observed under a microscope (Olympus). All processes were conducted strictly according to the manufacturer’s instructions.

## Results

### Characteristics of cells following induction

**Figure 1:**
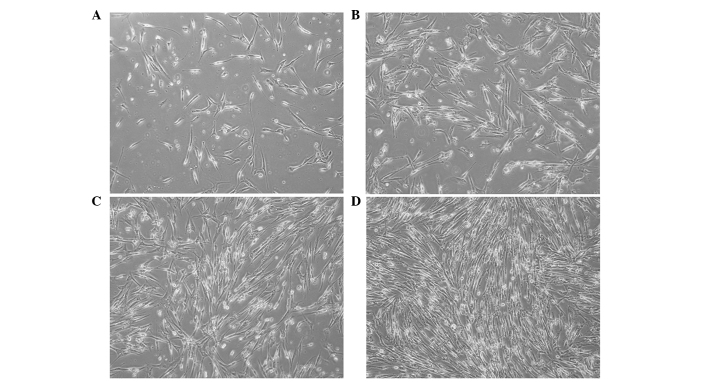
Morphological changes of the inducted cells after 1–4 weeks. (A) Spindle-shaped and irregular cells increased at the periphery of EBs after 1 week; (B) prominences in flanking cells were connected after 2 weeks; (C) connections between adjacent cells became apparent and formed an intercalated-like structure after 3 weeks; and (D) cells tended to be uniform after 4 weeks. EBs, embryoid bodies. Magnification, ×200.

Spindle-shaped and irregular cells increased in number at the periphery of EBs after 1 week (Fig. 1A). Prominences in flanking cells were connected after 2 weeks (Fig. 1B). The connections between adjacent cells became apparent and formed an intercalated-like structure in 3 weeks (Fig. 1C). The cells tended to be uniform after 4 weeks of treatment with 0.05 or 0.1 mg/ml ascorbic acid (Fig. 1D). The cell morphology did not change significantly following treatment with 0.01 mg/ml ascorbic acid; however, the majority of cells died under treatment with 0.2 mg/ml ascorbic acid.

### Optimal induction concentration

The effective concentration of ascorbic acid was 0.01–0.1 mg/ml and the inductive effect was dose-dependent. Comparisons in the conversion rates of the experimental and control groups revealed significant differences (P<0.01). Significant differences were also observed between the conversion rates at all time points (P<0.05) with the highest conversion rate at 3 weeks. Comparisons of the results for 0.1 mg/ml ascorbic acid with those of 0.05 and 0.01 mg/ml revealed significant differences (P<0.05). The results showed that the optimum concentration of ascorbic acid was 0.1 mg/ml (Table I).

### Expression of GATA-4 and cTnT detected by immunocytochemistry

**Figure 2:**
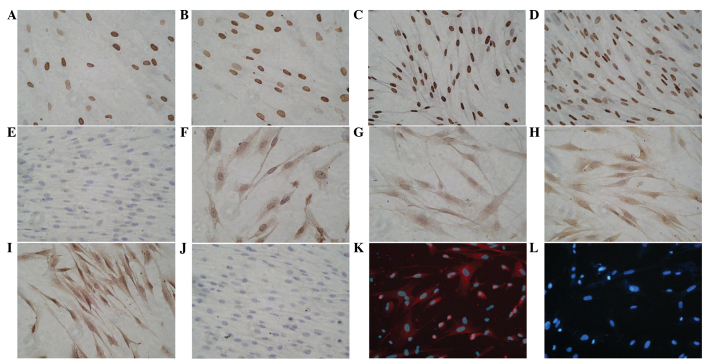
(A–E) Expression of the primordial myocardium-specific transcription factor, GATA-4, in hEGCs treated with 0.1 mg/ml ascorbic acid for 1–4 weeks (A–D) and in the control group (E) by immunocytochemistry staining; (F–J) expression of cardiac troponin T (cTnT) in hEGCs treated with 0.1 mg/ml ascorbic acid after 1–4 weeks (F–I) and in the control group (J) by immunocytochemistry staining; (K,L) expression of Cx43 by immunofluorescence in hEGCs treated with 0.1 mg/ml ascorbic acid for 2 weeks (K) and in the control group (L). Magnification, ×200. hEGCs, human embryonic germ cells.

Following treatment with 0.1 mg/ml ascorbic acid, the primordial myocardium-specific transcription factor, GATA-4, was stained positively in the differentiated cells after 1 week of induction; however, the positive cell number was low and brownish yellow granules were observed (Fig. 2A). An increased number of positive cells were stained 2 weeks following induction; the nucleus appeared oval-shape and stained brown in color (Fig. 2B). The expression of GATA-4 peaked after 3 weeks; the nucleus was long fusiform in shape and presented dark brown (Fig. 2C). The positive cell number decreased gradually and tended to be uniform after 4 weeks of induction (Fig. 2D). No expression of GATA-4 was observed in the normal tissue group (Fig. 2E).

Following treatment with 0.1 mg/ml ascorbic acid, cTnT expression was not observed until 2 weeks after induction and the expression was weak and the endochylema was stained brownish yellow (Fig. 2F and G). cTnT expression increased gradually over 3 and 4 weeks. cTnT was stained brown around the nucleus after 3 weeks of induction (Fig. 2H and I). No expression of cTnT was detected in the normal tissue group (Fig. 2J).

### Immunofluorescence of differentiated cardiomyocytes

Following treatment with 0.1 mg/ml ascorbic acid, the immunofluorescence assay showed that after induction for 2 weeks, numerous Cx43-stained cells emitted strong red fluorescence. The cells were polygonal and spindle-shaped (Fig. 2K). In the control group, the nuclei were stained blue with Hoechst 33342 (Fig. 2L).

### Rat model of AMI

**Figure 3:**
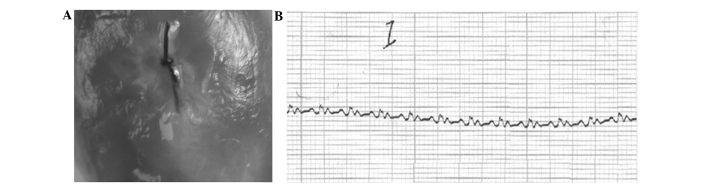
(A) Myocardial infarction in rats was observed under a dissecting microscope. (B) Changes consistent with myocardial infarction were observed in the rat model of myocardial infarction by electrocardiography.

After ligation of the left anterior descending artery in the surgery group, the distal end cardiac muscles of ligation were significantly pallid and their activity was weakened or disappeared at a certain range (Fig. 3A). The ECG showed limb lead ST-segment elevation to various degrees in the surgery group after 1–20 min of ligation and the pathological Q wave appeared (Fig. 3B). The sham surgery group were normal.

### Results of MAB1281 and GATA-4 immunohistochemistry

**Figure 4:**
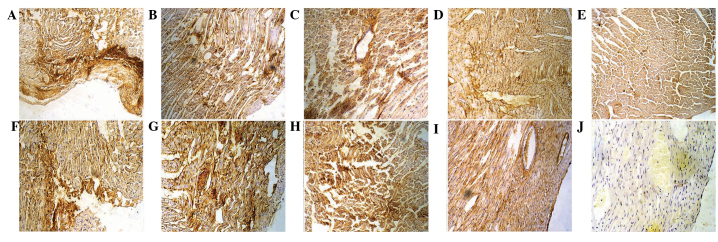
(A–E) Expression of MAB1281 in transplanted cells after 1 day and 1, 2 and 4 weeks (A–D) and in the control group (E) by immunohistochemical staining; and (F–J) expression of GATA-4 in transplanted cells after 1 day and 1, 2 and 4 weeks (F–I) and in the control group (J) by immunohistochemical staining. Magnification, ×100.

One day after transplantation, MAB1281-positive cells were distributed along the epicardium; the cells appeared round with a deep brown nucleus and were arranged in a disorderly manner (Fig. 4A). One week following transplantation, cardiac muscles in the infarcted zone showed necrosis and were disordered. There were MAB1281-positive cells around the myocardium, and cells were spindle-shaped and thin (Fig. 4B). Two weeks after transplantation, MAB1281-positive cells were arranged in the infarcted zone of the myocardium and were parallel to the cardiac muscles. They were long and spindle-shaped with a brown ovoid nucleus (Fig. 4C). Four weeks after transplantation, the majority of the cells in the infarcted zone were MAB1281 positive with a short column and brown nucleus, and the cardiomyocytes were aligned (Fig. 4D). The control group was negative for MAB1281 (Fig. 4E).

One day following transplantation, GATA-4-positive cells were dispersedly distributed under the epicardium and appeared round with a deep brown nucleus and a small amount of cytoplasm (Fig. 4F). One week after transplantation, GATA-4-positive cells were observed in the infarcted zone of the myocardium and along blood vessels; the cells were thin and spindle-shaped with a brown nucleus (Fig. 4G). Two weeks after transplantation, GATA-4-positive cells were arranged in the infarcted zone of the myocardium and were parallel with the cardiac muscles; the cells appeared long and spindle-shaped with a brown ovoid nucleus (Fig. 4H). Four weeks after transplantation, most cells of the infarcted zone were positive for GATA-4, had a short column and brown nucleus, and the cardiomyocytes were aligned in order (Fig. 4I). The control group was negative for GATA-4 expression (Fig. 4J).

## Discussion

Doetschman *et al* observed that, under certain conditions, ESCs rapidly differentiate into cardiomyocytes with a spontaneous rhythmic contraction; therefore, ESCs have become a useful tool for studying gene expression and heart function (9). It has been shown that differentiated cells *in vitro* are similar to cardiomyocytes *in vivo* in electrophysiological aspects and the cell cycle, which is indicative of broad prospects for the future of cardiomyocyte transplantation therapy (10). One of the major findings of the present study was that ascorbic acid served as a valuable inductor for the cardiac differentiation of hEGCs with high efficiency and efficacy *in vitro*. hEGCs differentiated into cardiomyocytes following treatment with ascorbic acid within a certain concentration range (0.05–0.1 mg/ml). The majority of cells died when the concentration reached 0.2 mg/ml due to the low pH and the precipitation of crystals. Therefore, hEGCs were treated with 0.1 mg/ml ascorbic acid and cardiomyocyte-like cells were obtained, which proliferated rapidly reaching a maximum at 10–12 days after induction. The immunofluorescence assay detected expression of Cx43 and cTnT in the cardiomyocyte-like cells.

A large reduction in the number of cardiomyocytes following myocardial infarction results in anatomical changes of the heart and remodeling of the left ventricle, ultimately resulting in heart failure. No type of reconstruction of the blood supply to the coronary artery or further medical treatment is able to prevent the loss of cardiomyocytes and left ventricular remodeling. However, transplantation of stem cells enables the regeneration of cardiomyocytes with normal function, thus inhibiting the formation of scar tissues and improving the cardiac function (11). A study has also shown the ability of hESC-derived cardiomyocyte transplantation to attenuate post-MI scar thinning and left ventricular dysfunction (12). In the present study, the mouse anti-human monoclonal antibody specific to nuclei (MAB1281), with a strong species specificity, was adopted to act as the tracer in immunohistochemistry. The present study showed that 1 day and 1, 2 and 4 weeks following transplantation the hEGCs gradually migrated into the myocardium, mostly to the infarcted zone with only a few migrating to the normal region in the heart. In the control group, rats were injected with the same volume of PBS and no expression of MAB1281 cells was detected.

Studies have shown that many tissue-specific transcription factors are associated with heart development, such as GATA-4, NKx2.5, Hey2, c-myb, Sox6 and Tbx (13–16), among which GATA-4 is the most closely associated. GATA-4 has been found to induce stem cell differentiation to cardiomyocytes, playing an important role in heart development at the early stage and it is also the earliest sign of cardiomyocyte progenitor cells. ESCs expressing GATA-4 may only differentiate to cardiomyocytes (17–20). In this study, hEGCs were transplanted into the infarcted zone of rats with myocardial infarction and 1 day and 1, 2 and 4 weeks later, positive expression of GATA-4 was observed in the progenitor cells, mainly in the nuclei. The number of GATA-4 positive cells increased with time. There was no expression in the control group. The data presented in the present study suggest that hEGCs survive after transplantation, then differentiate into cardiomyocyte progenitor cells. As the treatment was prolonged, the cells transplanted into the infarcted zone were arranged in an orderly manner, and were spindle-like, short and cylindrical in shape, which indicated that the cells began to differentiate to cardiomyocytes with a normal structure. Thus, the potential to treat myocardial infarction was demonstrated.

## Figures and Tables

**Table I:** Conversion rate of cardiac-like cells of hEGCs treated with ascorbic acid (%).

Groups	Concentration of ascorbic acid (mg/ml)	1 week	2 weeks	3 weeks	4 weeks
Control	0.00	0	0	0	0
Experimental	0.01	26.7±4.17^a^	37.5±0.65^a^	54.5±1.73^a^	38.0±3.27^a^
	0.05	41.3±2.34^a^	67.0±3.11^a^	80.6±3.35^a^	62.2±6.01^a^
	0.10	46.0±2.01^a^	71.3±1.36^a^	85.2±1.79^a^	77.4±1.49^a^
	0.20	0	0	0	0

a P<0.01, comparison between the experimental group and the control group and between all time points (P<0.05).

hEGCs, human embryonic germ cells.
